# Chinoidine and Capsicum in Intermittent Fever

**Published:** 1882-11-20

**Authors:** R. C. M. Page

**Affiliations:** New York


### CHINOIDINE AND CAPSICUM IN INTERMITTENT FEVER.

By R. C. M. PAGE, M. D., NEW YORK.

CASE I.—J. C——, iceman; born in Ireland; aged twenty-two, and single; has resided in New York city during the past fifteen years. Had a chill for the first time September 24, 1880. It came on about 10 a.m. and lasted an hour. It was well marked and was followed by the usual symptoms of headache, fever, vomiting and sweat. The chill returned on the 26th and 28th in the forenoon.

I saw him for the first time September 29, 1880. He had received no antiperiodic treatment, but had taken some "opening medicine" the day before, and his bowels had moved three times. General health good. Never drank to excess, but was in the habit of taking a glass of whisky occasionally.

*Treatment.*—Ordered three powders, each containing ten grains of powdered chinoidine and three grains of capsicum. To take one powder at bedtime, one at 6 a. m., and one at 12 m. next day. In order to make sure of his taking the whole powder, he was directed to shake it on his tongue from the paper, and to take a swallow of water afterward.

October 1st.—Chill has not returned. Ordered tr. ferri chloridi, in ten-drop doses well diluted, four times daily, and to continue the same for two weeks. I saw him three months afterwards, and he stated that there had been no return of the chills.

CASE II.—J. M——, laborer; born in Ireland; aged fifty-four, and married; has resided in New York city most of the time since 1849. About the middle of May, 1880, he went over to Hunter's Point, L. I., and remained there five weeks. During the last ten days of his stay there he had chills and fever for the first time in his life. I first saw him June 20th, following, and treated him with powdered chinoidine and capsicum. He was cured for the time, but was quite anæmic, and left off treatment too soon. As a consequence the chills returned.

September 27, 1880.—Patient applied to me again. Has had chills and fever off and on ever since I last saw him. During the past week he has had a chill every day. Bowels regular, but he is very anæmic.

*Treatment.*—Ordered six powders, same as in Case I. To take one powder before breakfast and at noon every day.

September 30th.—No return of chills. Ordered tr. ferri chloridi as in Case I. After two days the powders were repeated once and the tr. ferri chloridi pushed for a month. There had been no return of the chills when last seen six months ago.

CASE III.—K. M——, aged fifteen; born in the United States; resides in New York city. Had a chill for the first time August 20, 1880. She had them every other day for about ten days, when she took a bottle of "fever and ague cure," and got well, as she thought. I first saw her October 15, 1880. Had been having chills and fever off and on ever since the first attack. During the past week she had one every other day. Bowels constipated, and she is anæmic.

*Treatment.*—Ordered pil. aloes et ferri, to take one or two at bedtime as required to regulate her bowels, and powders of chinoidine and capsicum (gr. x. to j.)

October 18, 1880.—No chill since I last saw her. Ordered three more powders, and to take tr. ferrri chloridi as above described, and to continue it for a month.

There was no return of the chills up to February, 1882, when she was last seen.

The foregoing three cases are average descriptions of 140 cases of intermittent fever that have been treated by me during the past two years, of which I have any notes. Of these, 10 were treated with tinct. iodine without any appreciable benefit. The remaining 130 were treated with powdered chinoidine combined with capsicum, the dose of the former being always 10 grains, and the latter 1 to 3 grains, according to the age, sex and habits of the patient. Women and young people (not children) were given, as a general rule, only 1 grain of powdered capsicum. Men were given 3 grains, and beer-drinkers, of both sexes, were given 3 to 5 grains of powdered capsicum at each dose.

In only one case was vomiting produced by the powders. In large doses, however, and especially with children, chinoidine not unfrequently causes vomiting.

Of the 130 cases treated with chinoidine and capsicum the chills soon recurred in 9, evidently because the patients left off treatment too soon. Five were benefitted, though not cured—that is to say, the chills returned from time to time. This was doubtless due to their locality, occupation, etc., and nothing short of a change would effect a cure. Twenty-two cases were only seen once, but I believe that they were cured, or else they would have returned, since the majority of them were dispensary patients, having applied for treatment at the Northwestern Dispensary in this city.

Of 20 cases of intermittent fever recently treated by Dr. J. C. Mackenzie, of this city, in a similar manner, in only one case did the chills return within two weeks, and he had only applied once, having failed to return for iron. In one case the chills returned after the expiration of two months, but he had evidently taken the disease over again.

Dr. John H. Ripley recently treated a case of intermittent fever by giving large doses of quinine every day for a week, without success. He then gave 20 grains of chinoidine each day for three days, when the chill ceased to recur.

The powder of chinoidine should not be obtained by pulverizing the resinous mass simply, as the particles would soon coalesce again. But if it be evaporated to dryness and powdered in combination with some inert substance, as althea, it will then retain its form. Thus prepared it is about half the bulk of sulphate of quinine, and very much resembles in appearance the so-called dextroquinine. I have never given any other preparation of chinoidine than the powder, but see no reason why it should not be administered in the form of pills, capsules, etc.

Whatever be the value of chinoidine alone, it appears that in ten-grain doses, when combined with capsicum, it is nearly, if not quite, as good as quinine in breaking up ordinary cases of intermittent fever. Regulating the bowels, and following up the powder with iron in some form, preferably the tr. ferri chloridi, given frequently in moderate doses, and well diluted, appear to be necessary in order to effect a permanent cure.

With regard to chinoidine, Bartholow ("Mat. Med.") says: "When the mother liquor, left after the crystallization of the alkaloids of cinchona bark, is evaporated, a black residue is obtained which is called chinoidine. This contains amorphous quinia and cinchonia, and probably also quinidia and cinchonidia. It is a very efficient anti-periodic, and may be used with advantage as a substitute for quinia in doses about twice as large."

According to Nothnagel and Rossbach ("Handbuch der Arzneimittellehre," Berlin, 1880), chinoidine is "a resinous, dry mass, of a brown color, slightly soluble in water, but readily soluble in alcohol. This very cheap preparation is essentially a mixture of quinine, cinchonine, and resinous matters. The dose is considerably larger than that of quinine."

Prof. Binz, of Bonn, says: "When pure the action of chinoidine approximates that of quinine. The effect appears much more quickly, but passes off more rapidly than in the case of quinine, on account of its being more rapidly excreted" ("Grundzugs der Arzneimittellehre," Berlin, 1879.)

In view of the fact that quinine is very much more expensive than chinoidine, and that the latter can be used, when combined with capsicum, in as small doses as quinine, with equally good effect in the cure of most cases of ordinary intermittent fever, it would be a considerable saving of money to the laboring classes, in such cases, to buy chinoidine and capsicum instead of quinine —or even the other alkaloids of cinchona bark.—*N. Y. Med. Rec.*

